# Working Women in Large Towns

**Published:** 1890-12-06

**Authors:** 


### Working Women in Large Towns.

### XVII.—AIDS FOR WORKING WOMEN.

Some of Mr. Walter Besant's readers have made it a serious ground of complaint against him that in his tales of work-girls, their difficulties and temptations, he has entirely ignored the various efforts which have been made for their welfare by the Church, and by other philanthropic agencies. We ourselves are not disposed to join the ranks of his censors, feeling that he understands the rules of his art far better than we do, and seeing that an artist must needs choose out from a mass of materials those, and those alone, upon which he can work, putting aside many more which, valuable as they are, can only properly be used by the author of a treatise. But as we ourselves partake of the nature of Dry-as-dusts rather than of artists, it now becomes our duty—and, we may add, our pleasure—to inquire what has already been done for our working women in the way of help.

We do not propose to speak of what is ordinarily known as "charity." That, no doubt, has its uses—in exceptional times and circumstances; but the melancholy fact remains, that this kind of charity too often tends to perpetuate, rather than to relieve, distress. What is wanted is something which shall raise the economic, social, and moral condition of our working women; and any efforts which seem to tend in this direction need all the encouragement and help that can be given them. To some extent we have already indicated the lines upon which—in our opinion, at least—those who wish to help working women may most profitably proceed; it now remains for us to give definite information as to what has been, or is being, done.

*Trades Unions.*—Those who have seriously gone into the questionare unanimously agreed, we believe, that combination among female workers will be productive of good in many ways, not only to the women themselves, but to men also, who now find the unorganized labour of the other sex an important factor in the reduction of wages in trades where both men and women are employed. It is distinctly more difficult to organize women than men; they have less pluck, energy, and *esprit de corps*, and, if they work at home, are very hard to get at. Nevertheless, there are several very successful unions among women of various trades, and it is to be hoped that many more will be started, under the auspices of one or other of the two societies which have been formed for this purpose—the Women's Trades Union Provident League, and the Women's Trades Association. The former of these, of which the Women's Trades Association is a recent offshoot, has been doing good work now for fifteen years. The unions which it has formed not only provide sick and out-of-work pay, but look after the interests of their members generally, aiming to secure for them sanitary workrooms, reasonable hours, proper treatment at the hands of employers and foremen, and, where it is possible, better wages. This they endeavour to do, not by fighting (though a strike may occasionally be found necessary), but rather by binding the workers together, and saving them from the cruel results of unchecked competition.

The oldest union is the Female Bookbinders', which numbers about two hundred members. Among other London unions are the Matchmakers', the Cigar-makers', and the Fulham Laundresses', which has four branches, and is in a very flourishing state. A union has also been started for East-end tailoresses; it is very small at present, but its numbers are increasing. As space is limited, we must pass over several others which claim our notice, and go on to those in other English towns, choosing out for special mention the Nottingham Cigar-makers' Union; the Liverpool Cigar-makers', Bookbinders', and Tailoresses'; the Leeds Tailoresses'; and two societies in Oxford. In Scotland, again, where the women seem to be more self-reliant and energetic than their English sisters, there are several large unions. Not only do some men's unions admit female members, but others have given sympathy and help to new and struggling unions for women only; and we cannot doubt that the movement will attract more attention in days to come than has yet been bestowed upon it. The unions, when once fairly started, are absolutely self-supporting, one of their great objects being to develop self-reliance among women; but expenses must be incurred in the work of establishing them, and it is to be hoped that Lady Dilke's eloquent appeal (in the January number of the *New Review*) will be responded to, both by those who can give money and those who will give practical help. "There are many directions," so runs the report of the League, "in which we desire to be at work, but are held back by want of money."

*Co-operative Workrooms.*—These may be started by the workers themselves, or by those who wish to help them, and are useful both directly, by giving a fair price for work done in them, and indirectly, by tending to advance wages generally. Mr. Walker's experiment in this way among the shirtmakers was noticed in a former article; and there are at least two other Co-operative Needlewomen's Societies, their respective workrooms being in Carteret Street, Westminster, and Brooke Street, Holborn. We have made careful inquiries with regard to the latter, and believe it to be doing excellent work, both as regards its *employées*, who receive a fair, though not a high, wage, and sit in a pleasant, airy workroom; and also as regards the shirts and collars, and various kinds of underclothing for ladies and children, upon which these women are engaged. If ladies wish to have their work done "cheaply," *i.e.*, at a price which will ensure the poverty and privation of the worker, this is not the place to come to; on the other hand, the prices charged are not "high," and the work is well done. Many more poor needlewomen might be helped, if these co-operative workrooms were more patronised, and there seems to be no reason why such undertakings should not meet with a considerable amount of success. Only—they must be managed by persons of real business capacity; in trade, as in everything else, philanthropy needs to be supplemented by sound common-sense and practical knowledge; otherwise, it is sure not to succeed.

*(To be continued.)*

